# Update on Respiratory Fungal Infections in Cystic Fibrosis Lung Disease and after Lung Transplantation

**DOI:** 10.3390/jof6040381

**Published:** 2020-12-21

**Authors:** Sabine Renner, Edith Nachbaur, Peter Jaksch, Eleonora Dehlink

**Affiliations:** 1 Division of Pediatric Pulmonology, Allergology and Endocrinology, Department of Pediatrics and Adolescent Medicine, Associated National Center in the European Reference Network on Rare Respiratory Diseases ERN-LUNG and the European Reference Network on Transplantation in Children, ERN TRANSPLANT-CHILD, Medical University of Vienna, 1090 Vienna, Austria; sabine.renner@meduniwien.ac.at (S.R.); Edith.nachbaur@meduniwien.ac.at (E.N.); 2 Division of Thoracic Surgery, Medical University of Vienna, 1090 Vienna, Austria; Peter.jaksch@meduniwien.ac.at

**Keywords:** ABPA, fungi, respiratory exacerbation, antifungals

## Abstract

Cystic fibrosis is the most common autosomal-recessive metabolic disease in the Western world. Impaired trans-membrane chloride transport via the cystic fibrosis transmembrane conductance regulator (CFTR) protein causes thickened body fluids. In the respiratory system, this leads to chronic suppurative cough and recurrent pulmonary infective exacerbations, resulting in progressive lung damage and respiratory failure. Whilst the impact of bacterial infections on CF lung disease has long been recognized, our understanding of pulmonary mycosis is less clear. The range and detection rates of fungal taxa isolated from CF airway samples are expanding, however, in the absence of consensus criteria and univocal treatment protocols for most respiratory fungal conditions, interpretation of laboratory reports and the decision to treat remain challenging. In this review, we give an overview on fungal airway infections in CF and CF-lung transplant recipients and focus on the most common fungal taxa detected in CF, *Aspergillus fumigatus*, *Candida* spp., *Scedosporium apiospermum complex*, *Lomentospora* species, and *Exophiala dermatitidis*, their clinical presentations, common treatments and prophylactic strategies, and clinical challenges from a physician’s point of view.

## 1. Introduction

Cystic fibrosis (CF) is the most common rare genetic disease in Europe and the United States with an incidence of 1:3500 [1]. The autosomal-recessive genetic disorder caused by mutations on the long arm of chromosome 7, of which more than 2000 mutations, 360 of them disease causing have been identified to date [2], leads to dysfunction of the cystic fibrosis transmembrane conductance regulator protein (CFTR-protein) in epithelial cells (e.g., in the sweat glands, the respiratory system, and the digestive system). This results in impaired salt- and bicarbonate secretion. In the respiratory system, the lack of chloride ion secretion causes a highly increased mucus viscosity, hampering mucociliary airway clearance. CF mucus also provides ideal conditions for bacterial and fungal growth. CF lung disease is characterized by a chronic suppurative cough, recurrent infectious exacerbations, and eventually, chronic colonization with bacterial pathogens such as *Pseudomonas aeruginosa* or *Staphylococcus aureus.* Progressive decline in lung function and irreversible structural changes of the lungs culminate in end stage lung disease with chronic respiratory failure.

In the past decades, advances in antibiotic treatment regimens have greatly improved the survival of CF patients. Daily chest physiotherapy and targeted antibiotic treatment of respiratory infections have become hallmarks of CF therapy [3,4]. In recent years, CFTR modulators, compounds directly targeting the production and/or function of the malfunctioning CFTR protein to rectify the aberrant ion transport, have shown impressive effects on lung function, pulmonary exacerbation rates, nutritional status, general condition, and disease progression in CF patients with eligible CFTR mutations [5,6,7,8]. These active substances will likely change CF clinical care in the near future since they tackle the key defect, the faulted CFTR-protein, rather than treating CF symptoms and consequences of organ damage. With more and more countries including CF in their neonatal screening programs, the introduction of CFTR modulating therapies early in life poses a real chance to prevent irreversible damage to the lungs and other organs such as the pancreas and the liver.

At present though, the management of infective pulmonary exacerbations and chronic bacterial airway colonization remains key in the treatment of CF lung disease. Aside from bacterial species, CF airway samples are often positive for yeasts and molds, but in contrast to bacterial infections, our knowledge about the clinical significance of fungi in CF lung disease is rather limited.

From the clinician’s angle of view, the incidence of mycosis in CF patients seems to be on the rise, and the diversity of fungal species on microbiology reports from patient samples appears ever expanding. It is up for debate whether there is a true increase in the incidence of emerging pathogens or whether other factors such as better diagnostic and raised awareness, not at least due to rising numbers of immunocompromised patients (e.g., lung transplant recipients), add to this impression. 

In this review, we aim to give a basic overview on fungal airway infections in CF. We will review clinical aspects of pulmonary mycosis in CF and the clinicians’ dilemma with regard to treatment decisions when fungi are reported in a patient’s airway sample. We will focus on the fungi most commonly detected in CF airway samples, *Aspergillus fumigatus*, *Candida* spp., *Scedosporium apiospermum complex*, *Lomentospora* species, and *Exophiala dermatitidis* their clinical presentations, if any, common treatment strategies, and clinical challenges from a physician’s point of view. We cannot cover all aspects relevant to the emerging field of fungal respiratory infections in CF (e.g., the complex host–pathogen interactions, physiological mycobiota–microbiota interactions, immunological phenomena, or details on diagnostic methods). These topics have been reviewed elsewhere [9,10,11,12].

## 2. Epidemiology

Large studies have shown that fungal colonization is very common in CF, but prevalence rates of fungal taxa vary greatly between studies (Table 1).

A retrospective analysis of over 25,000 samples from more than 600 CF patients by the German National Reference Center revealed that around 75% of CF patients were colonized by yeasts, mainly *Candida* spp., with a majority of *Candida albicans*, and about 35% were positive for *Aspergillus* spp. Other filamentous fungi such as *Exophiala dermatitidis* and *Scedosporium/Lomentospora* complex isolates were found in around 4% of patients, with up to 10% in some CF centers. Authors have reported that between 2009 and 2013, the epidemiology of fungal species remained largely unchanged over the study period. Environmental exposure to fungi and consecutive airway colonization may fluctuate with seasons, but in this study, there were no annual or seasonal variations in the detection rates [13]. In contrast, an analysis of the United Kingdom CF Registry showed a rise in the prevalence of *Aspergillus* spp. from 6.5% in 2007 to 13.6% in 2012. Additionally, rare fungi (e.g., *Scedosporium* spp.) were detected slightly more often in 2012 (0.68%) than in 2007 (0.07%) [17]. It has to be noted though that the use of varying culture protocols in studies and nonuniform diagnostic techniques across CF centers partaking in registries makes direct comparison of data complicated. We know that in CF patients, co-colonization of bacteria with faster growth rates, like *Pseudomonas aeruginosa* and *Staphylococcus aureus*, can mask the presence of more slowly growing fungi and mycological protocols using specific pretreatment protocols and fungal selection media are inevitable. A recent prospective study from the Netherlands confirmed that the introduction of modified fungal culture protocols led to an increase in detection rates and diversity of fungal species. Using mucolytic pre-treatment, a larger sample volume (100 microl), specific fungal culture media (Sabouraud agar; SAB, Medium B+, Scedosporium selective agar; SceSel+ and Dichloran-Glycerol agar; DG18), and a longer incubation time of three weeks, the authors detected *Aspergillus fumigatus* in 55.9%, *Scedosporium* spp. in 1.1%, and *Exophiala* spp. in 1.5% of CF sputa compared to 33.8%, 0.9%, and 0.1% in a retrospective cohort [18]. In a 3-year prospective multi-center study in French CF-centers, defined diagnostic protocols were compared for their diagnostic yield. Mucolytic pretreatment and cultivation on four culture media (Sabouraud-chloramphenicol (SAB27), erythritol-enriched (ERY27), chromogenic (CAN37), and cycloheximidine-enrched (ACT37) media) for 16 days yielded the best results. *Candida albicans* was detected in 58.8% of patients and 35.4% had *Aspergillus fumigatus* in their samples [14]. A prospective study from the United States using selective mycological media for CF sputa including Sabouraud dextrose agar with gentamicin (SDA), inhibitory mold agar (IMA), and brain heart infusion (BHI) agar with chloramphenicol and gentamicin revealed prevalence rates of 40.8% for *Aspergillus* spp., 5.2% for *Scedosporium*, and 4.7% for *Exophiala* [19]. In a retrospective cross-center analysis across nine European CF centers, Schwarz and colleagues supported the findings from the U.S., Germany, and the Netherlands with regard to dispersed prevalence rates. They too identified large differences between centers with regard to fungal diversity and prevalence of specific fungal taxa, further underlining the impact of different culture conditions and -duration, and diagnostic protocols [15]. To improve and harmonize the detection and the clinical knowledge of fungal infections in CF, the working group on fungal respiratory infections in cystic fibrosis within the International Society for Human and Animal Mycology (ISHAM) since 2006 has been working on developing and standardizing mycological culture protocols and nonculture-based diagnostics—techniques that have further broadened the spectrum of fungi species found in CF respiratory samples, but with often unclear clinical significance—and linking mycologists with clinicians to improve patient management [12].

Aside from diagnostic techniques, a number of factors are being discussed to impact the prevalence and diversity of fungi. In vivo, bacteria and fungi co-colonize CF lungs, hence it is fair to assume that they compete and interact. Research on the interplay between fungi like *Aspergillus fumigatus* and the lung microbiota, in particular *Pseudomonas aeruginosa*, has been emerging, and both inhibitory as well as stimulating effects on growth and virulence have been reported in vitro and in ex vivo models [11,24]. Geographic and climate factors may influence environmental exposure and consecutive fungal infections. A European multi-center study revealed a north to south increase in the prevalence of *Scedosporium*, while *Aspergillus fumigatus*, *Cladosporium*, and *Penicilium* were more prevalent in centers from Northern Europe [25]. An Australian study implicated that man-made environments affect the abundance of fungi [26]. Authors found a higher prevalence of *Scedosporium* spp. in soil samples from urban environments compared to samples from semirural and rural areas, which could explain the comparatively high rates of *Scedosporium* infections and colonization in Australia [20]. Similar to the German study, there was no evidence of seasonality during repeated sampling. Lifestyle factors such as pet ownership as a potential source of mold exposure has been associated with allergic broncho-pulmonary aspergillosis ABPA in a German study [27]. With age, progressive lung damage, and declining forced expiratory volume in 1 second (FEV1), the risk of fungal infestation seems to increase, but CF care itself may also play a role. In their study across nine European CF centers, Schwarz et al. discovered considerable heterogeneity with regard to infection control measures and hygiene recommendations to patients, which could affect regional differences in pulmonary mycosis. In our experience, patients and their families are advised to reduce possible sources of environmental exposure to fungi as much as possible (e.g., to avoid damp flats/houses, organic waste bins, indoor plants, or animal cages). Of note, high loads of airborne *Aspergillus fumigatus* and aspergillosis outbreaks in lung transplant recipients have been reported during heavy construction works in hospitals, which can be prevented by high efficiency particulate air filtration (HEPA) [28,29,30].

An emerging number of studies have discussed a role of CF treatment as a risk factor for recovering fungi, namely frequent courses of antibiotics, chronic suppressive inhaled antibiotics for chronic airway infections, inhaled steroids, and oral macrolides [31,32,33,34]. Findings are not uniform though, and both increased and decreased rates have been reported. It could be argued that these observations simply reflect the severity of CF lung disease, which is associated with the risk of recovering fungal infections. CFTR modulator therapies hold promise to improve lung health and reduce airway infections. CFTR modulator therapy with ivacaftor for the gating mutation G551D was associated with a reduction in detection rates for *Aspergillus* spp. and *P. aeruginosa* compared to before treatment [35]. 

A particularly vulnerable population with regard to recovering fungal infections is CF patients under immunosuppressive therapy after lung transplantation. This aspect will be covered later in this article. 

In summary, the epidemiology of fungal species in CF lung disease is multifactorial and data have to be interpreted with caution. Further complicating matters for clinical practice, the relevance and pathogenicity of most fungi are variable and poorly understood, and most clinical manifestations lack clear diagnostic criteria. As a consequence, the decision to treat can be challenging. Clinical patterns of fungal infections in CF are summarized in Table 2.

In contrast to the epidemiology of fungal taxa detected in CF airway samples and the prevalence of ABPA, which is collected in the large CF registries, the prevalence of fungal bronchitis and pneumonia are not clearly known. In the absence of consensus criteria for the latter two, experts in the field have proposed a combination of empiric diagnostic criteria, which we found very useful in discriminating a “highly probable” pulmonary fungal infection from simple colonization [36]:

1. Increased sputum production.
2. Multiple isolations of the same fungal species from sputum or bronchoalveolar lavage, BAL) (at least two culture-positive samples in six months).
3. Pulmonary infiltrate(s) on chest CT scan or X-ray.
4. Treatment failure with antibiotic therapy (two and more antibiotic treatments, duration two or more weeks).
5. Unexplained lung function decline.
6. Exclusion of new/other bacteria.
7. Exclusion of allergic bronchopulmonary aspergillosis.

Tracy and colleagues amended these criteria and introduced “minor criteria” such as elevated fungal specific IgG, positive galactomannan in sputum or BAL, and cytology/histology findings in BAL and biopsies [37].

The lack of consensus criteria for most clinical manifestations of fungal infections also hampers the design of prospective interventional clinical trials and the development of evidence based treatment guidelines. Except for ABPA, no consensus recommendations for the treatment of fungal infections in CF have been agreed on yet. Active substances most commonly used are azoles (oral itraconazole, oral or intravenous posaconazole and voriconazole), polyenes (nebulized or intravenous amphothericin B), and echinocandins (intravenous caspofungin, micafungin, and anidulafungin). The choice of antifungal agents and duration of treatment are individual, and depend on the fungal species detected, its antifungal resistance, and the clinical status of the patient. Treatment courses can be between two weeks to up to more than six months and usually consist of single agent therapy, but can be escalated to combinations of up to three agents in highly resistant fungi or severe infection (reviewed in [36]). We will summarize common practices and recommendations in the paragraphs on the respective fungi.

## 3. *Candida* spp.

Colonization with yeast is very common in CF, especially in throat swabs, with *Candida albicans* being the taxa most frequently isolated (up to 62% [16]). Other species commonly found are *Candida dubliniensis*, *Candida glabrata*, and *Candida parapsilosis* [13,14,15,16,36]. They are only rarely detected in broncho-alveolar lavage samples though, which raises doubts about their clinical significance as pathogens versus simple oral cavity colonizers. The decision to treat is made individually, guided by the criteria for “highly probable” pulmonary fungal infection.

While *Candida* spp. does not seem to cause pulmonary exacerbations, a recent retrospective analysis over a period of 16 years revealed that colonization with *C. albicans* or *A. fumigatus*, but in particular *Candida dubliniensis* in sputum, was associated with a decline in lung function [16]. *Candida albicans* also plays a role in severely ill CF patients who need central venous access for frequent antibiotic treatments. These patients are at risk of *Candida* sepsis, which not only requires systemic antifungal treatment to prevent complications such as endocarditis, but often means the loss of central venous access for treatment.

In 2019, *Candida auris*, a very resistant Candida species that typically colonizes the ear canal [38], was first detected in the sputum of a CF patient who was receiving long-term posaconazole treatment for *Aspergillus* colonization [39]. *Candida auris* has not been described in CF lung disease, but may be worth considering in patients with unexplained lung function decline under long-term anti-fungal treatment.

## 4. *Aspergillus* spp.

As elaborated on above, *Aspergillus* spp. are isolated in around 40% of CF patients. The most common species is *Aspergillus fumigatus*, the rarer ones are *Aspergillus flavus*, *niger*, *clavatus*, *nidulans*, or *terreus*.

In clinical practice, *Aspergillus* bronchitis seems to be the most frequently observed clinical entity, characterized by pulmonary exacerbations that fail to respond to antibiotic treatment, but clinical and spirometry improvement with antifungal therapy [40]. A diagnosis of *Aspergillus* bronchitis is supported by the presence of *Aspergillus fumigatus* in sputum, galactomannan in sputum and the presence of specific *Aspergillus fumigatus* IgG in the absence of specific IgE [41]. Combining these clinical and laboratory criteria for *Aspergillus* bronchitis, Brandt et al. reported a prevalence of only 1.6% in a retrospective cohort and 9% in a prospective study. Antifungal treatment for two to six weeks reduced the clinical signs of exacerbation and improved lung function [42].

For the treatment of aspergillosis, in particular for invasive disease, voriconazole has been recommended as first-line choice. Other preferred regimens are amphotericin B and isavuconazole. Posaconazole can be considered as an alternative [36,43,44]. In rare cases, *Aspergillus* colonization can give rise to pulmonary aspergilloma in severe CF lung disease [45].

## 5. Allergic Broncho-Pulmonary Aspergillosis (ABPA)

ABPA is a common complication of CF lung diseases and should be considered in any patient with clinical deterioration not responding to antibiotic therapy. Incidence increases with age and with great variability between countries and geographic regions. In Europe, ABPA affects around 2–14% of CF patients [46].

The immunologic mechanism is quite well characterized: ABPA is an IgE-mediated allergic reaction to *Aspergillus fumigatus* in the bronchial system, similar to asthma, with bronchial constriction and allergic airway inflammation [47,48].

ABPA is a severe clinical complication of CF and has a negative effect on lung function and overall survival [49]. Symptoms comprise reduced general condition, dyspnea, and increased cough and sputum, typically red-brown in color. Diagnosis can be difficult, since these symptoms are not specific and not exclusive to ABPA.

To make a diagnosis of ABPA, the ISHAM ABPA working group proposes the presence of a combination of diagnostic criteria [50]:

Both obligatory criteria:

1. Skin prick test to *Aspergillus fumigatus* positive or the presence of specific IgE to *Aspergillus fumigatus*.
2. Elevated total IgE >1000 kU/L.

and two of the following criteria:

1. Presence of precipitating *Aspergillus fumigatus* IgG antibodies.
2. Eosinophilia of more than 500 cells/microl (in steroid naïve patients).
3. Radiographic pulmonary opacities consistent with ABPA.

In a recent study, lowering the serum total IgE cut off to >500 IU/mL excluding the skin test and using CT thorax instead of conventional chest radiograph, further improved the diagnostic performance [50,51]. Additional markers that can support a diagnosis of ABPA comprise bronchodilator reversibility (BDR), fractioned exhaled nitric oxide (FeNO), and eosinophilic cationic protein (ECP).

ABPA is treated with a combination of antifungal agents to clear Aspergillus and anti-inflammatory therapy to suppress airway inflammation and immunological pathways [48]: as an antifungal, itraconazole is used most commonly, but monitoring of trough levels is indicated because absorption shows great inter-individual variability [48]. In addition, there is increasing evidence of itraconazole resistance of *Aspergillus fumigatus* (8% to 20% [52,53]). Voriconazole and posaconazole can be used as alternatives. Anti-inflammatory treatment consists of prednisolone for 6–12 weeks, followed by tapering down. Instead, some authors prefer steroid pulse therapy [54]. Patients under ABPA treatment need to be closely monitored, not only with regard to achieving therapeutic itraconazole levels. Azoles can trigger derangement of liver enzymes and can cause problems due to drug–drug interactions (e.g., with CFTR-modulators through the CYP450 pathway). Long-term steroid treatment comes with a range of adverse effects like triggering or deranging CF-related diabetes mellitus, adrenal suppression, and the risk of acquiring non-tuberculous mycobacteria. As an alternative to steroids, anti-IgE monoclonal antibody (mAb), omalizumab, has gained attention as a more targeted treatment of the allergic reaction with a more favorable safety profile than steroids [55,56]: Omalizumab is administered every 2–4 weeks subcutaneously. Omalizumab is more expensive than steroids and its use for ABPA is off-label, but it has the potential of a steroid sparing effect. Of note, a recent Cochrane Review concluded that there was not enough evidence in support of the efficacy and safety of omalizumab therapy for the treatment of ABPA in CF [57]. Other therapeutic monoclonal antibodies that target type 2 inflammation pathways, anti-IL-5 (mepolizumab) [58], and IL-4/IL13 mAb (dupilumab) [59] have shown positive effects in case series. Regardless of which therapeutic agents are used, total treatment duration is usually several months [60], which even without the frequent side effects and treatment complications poses challenges to the patients’ treatment adherence.

## 6. Scedosporium Apiospermum Complex, Lomentospora Species, and Exophiala Dermatitidis

Slowly growing filamentous fungal taxa *Scedosporium* complex (*S. apiospermum* sensu stricto, *S. boydii*, *S. aurantiacum*, *S. minutispora*, and *S. dehoogii*), *Lomentospora prolificans* (formerly S. prolificans), and *Exophiala dermatitidis* are increasingly being recognized as relevant to CF lung disease. With up to 10% of CF patients colonized [21], *Scedosporium* and *Lomentospora* species are the most common fungal taxa after *Aspergillus* spp. *E. dermatitidis* has been reported with a prevalence between 2% and 18% [22,23,61]. In a recent study using molecular diagnostics, *Scedosporium* spp. and *Exophiala dermatitidis* were the most abundant taxa in CF patients with fungal bronchitis [62]. Co-colonization with *Aspergillus* spp. is frequently observed [20], whilst increased as well as decreased co-colonization with mucoid Pseudomonas aeruginosa has been reported [20,21]. Younger age and a history of ABPA have also been associated with *Scedosporium/Lomentospora* colonization [63]. In a large study using data from the Dutch CF-registry, comprising data from 1312 CF patients, *Scedosporium* was found in 7% of CF patients, and was associated with severe CF-genotype, inhaled antibiotics, and CF-related diabetes, whilst *for E. dermatitidis*, a positive association was found with older age, female gender, and *Aspergillus* spp. co-colonization. *E. dermatitidis* was detected in 2% of patients [23]. In particular, in the immunocompromised host after lung transplantation, *Scedosporium* species and *Lomentospora prolificans* colonization can progress to life threatening invasive disease. After *Aspergillus* spp., these two taxa are the second most prevalent causes of invasive fungal disease in transplant recipients, and airway colonization is a contraindication for lung transplantation in many centers [64].

*Scedosporium/Lomentospora* pulmonary exacerbations are difficult to treat due to high antifungal resistance in *Scedosporium* and multi-drug resistance in *Lomentospora*. In general, voriconazole has been recommended as first-line choice [65,66]. In severe disease or failure to respond, combinations of two or even three different antifungals based on susceptibility testing have been used successfully (e.g., voriconazole with amphotericin B or echinocandins for *S. apiospermum* and *L. prolificans* infections, or oral triazoles in combination with an intravenous echinocandin and inhaled amphotericin B for infections with *Scedosporium* species) [36,67].

## 7. Pulmonary Fungal Infections in Lung Transplant Recipients

In recent years, allogeneic lung transplantation has become an established therapy for eligible patients with end stage lung disease. Worldwide, approximately 100 pediatric and 4500 adult lung transplantations are performed each year [68].

CF lung disease is the most frequent underlying disease in children, followed by idiopathic pulmonary arterial hypertension (IAPH) and rare diseases like alveolar proteinosis or idiopathic pulmonary hemosiderosis. In adults, the most common indications are chronic obstructive pulmonary disease (COPD), idiopathic pulmonary fibrosis (IPF), and CF.

In particular, in the first year after solid organ transplantation, infections including invasive fungal disease represent the major cause of morbidity and mortality [69,70,71]. Among fungal pathogens, invasive fungal infections with *Aspergillus fumigatus* are the main threat, isolated in three to 44% of cases [72,73,74] with considerable mortality rates of 41 to 51% [75], followed by infections with *Candida* spp. (23%) [76], most commonly *Candida albicans*, *Cryptococcus* spp. (2%) [74], Mucorales/Zygomyzosis (up to 3%) [74,76], endemic mycosis (*Histoplasma*, *Blastomyces* spp., *Coccidioides*, 1% [74], *Scedosporium* (3.5%) [76] and *Pneumocystis jirovecii* (2%) [74], and *Exophiala dermatitidis* (case report) [77].

Respiratory tract manifestations of fungal infection can be fungal pneumonia, invasive disease or local infection of the anastomoses. Invasive pulmonary fungal infections (PFI) with *Aspergillus* spp. typically present with fever, pleuritic chest pain, dyspnea, cough and hemoptysis can disseminate to other organs and progress to fungemia and systemic infection. A distinct entity in lung transplanted patients is Aspergillus tracheobronchitis (ATB). It usually affects the bronchial anastomosis, predominantly within the first three months post transplantation and predisposes to local bacterial infections due to disruption of the mucosal barrier integrity [78].

A broad range of risk factors for pulmonary fungal infections after lung transplantation have been proposed (reviewed in [79]). Factors related to the lung allograft, like environmental exposure and infection of the lung allograft [80], increased donor age [81], anatomic abnormalities, and physiologic characteristics of the transplanted lung such as impaired mucociliary clearance, blunted cough reflex, denervation injury, and bronchial anastomotic complications [82] could predispose to a higher risk of invasive fungal infections. Host-related risk factors include older age, augmented immunosuppressive medication [81], cytomegaly virus infection, A2 grade allograft rejection, repeated allograft rejections, and single lung transplantation [83] airway colonization with *Aspergillus* spp. [81,83]. Below, we elaborate on the role of fungal airway colonization in fungal disease after lung transplantation.

## 8. Fungal Airway Colonization, Pulmonary Fungal Infection (PFI), and Bronchiolitis Obliterans Syndrome (BOS) after Lung Transplantation

With CF being a leading cause for lung transplantation, and a large number of CF patients being chronically colonized with fungi, one would assume that CF patients are at high risk of pulmonary mycosis after lung transplantation. The number of studies on this matter is limited though and study populations are often heterogeneous and small in numbers, and the results are not univocal. In a large retrospective multicenter study in 555 pediatric lung transplant patients from 1988 to 2005, Danzinger-Isakov et al. reported a prevalence of PFI of 10.5% in the first year after transplantation, with a mortality rate of 38%. The vast majority occurred in the first six months after transplantation, and *Aspergillus* and *Candida* were the most common fungal taxa identified. These authors found that, among other factors, pre-transplant colonization was predisposed to post-transplant PFI [84]. In a smaller cohort, Liu et al. retrospectively analyzed 55 pediatric lung transplant recipients from a single center and reported a pre-transplant fungal colonization rate of 53% (29/55), and a post-transplantation colonization rate of 60% (33/55). Eleven patients (20%) developed pulmonary fungal disease, but there was no significant association of colonization with pulmonary fungal disease [85]. In a subsequent prospective study including 59 pediatric lung transplant patients, 29 of whom had CF, Ammerman et al. found that CF was associated with pre-transplant fungal colonization, but neither pre- nor post-transplant colonization were associated with PFI with the same fungal organism. The spectrum of fungal species detected was similar to the general CF population, dominated by *Candida* and *Aspergillus*. A total of 10/59 (17%) patients fulfilled the criteria for PFI, which was defined as the presence of all of the following: a positive culture, symptoms suggestive of pulmonary fungal infection, radiological changes or locally visualized purulent sputum without other cause, a positive histopathology or cultured organism from sterile tissue. No patient died of fungal infections [71].

A recent retrospective international cohort study in 900 adult lung transplant recipients with miscellaneous underlying diseases revealed that despite routinely used extensive antifungal prophylaxis, invasive *Aspergillus* infection was still associated with higher mortality. No association of a diagnosis of CF or pre-transplant colonization with *Aspergillus* spp. and invasive pulmonary infection was observed though, but across all subjects and all underlying diagnosis, colonization with *Aspergillus* spp. within one year after transplantation was a risk factor for invasive *Aspergillus* spp. infection [83]. In summary, compared to studies from before the era of antifungal prophylaxis, these more recent studies implicate that although pre-transplant fungal airway colonization is common in CF lung transplant recipients, it does not seem to be a significant risk factor for pulmonary fungal infection or death.

Aside from PFI, fungal colonization has been attributed as a risk factor for Bronchiolitis obliterans syndrome (BOS), a disease of the small airways leading to chronic allograft failure. BOS is part of the spectrum of chronic lung allograft dysfunction (CLAD) and is responsible for >40% death after the first year of lung transplantation [70,86]. The etiology of BOS is multifactorial, and data on the role of fungi, in particular *Aspergillus* spp., are contradictory. In a study on two large adult cohorts in the United States, colonization with small, but not large, conidia *Aspergillus* species with a diameter of ≤3.5 μm was a risk factor for BOS and death. Authors attributed this finding to the higher likelihood of colonization of the small airways, the site of BOS [87]. However, in another recent large retrospective study, no association between *Aspergillus* colonization, neither small nor large conidia, and the development of BOS was found [88].

## 9. Post-Transplant Antifungal Prophylaxis

Due to the high risk of post-transplant fungal infection and its adverse effects on survival, antifungal prophylaxis has become common practice in the care of lung transplant recipients. Prophylaxis strategies are heterogeneous and mostly center-specific though, the duration of prophylaxis ranges from six to 12 months post-transplant, some centers recommend universal strategies, independent of colonization status and diagnosis, others use targeted or empiric prophylaxis and pre-emptive treatment whenever fungi are detected in airway samples. Single-agent prophylaxis is used as well as combinations of inhaled liposomal amphotericin B and oral triazoles [79]. A recent published survey across 44 United States adult lung transplant centers revealed that 97.5% of centers used post-transplant prophylaxis, 90% of centers advocating universal prophylaxis with nebulized amphotericin in combination with triazole agents for six months or less, and four centers also used pretransplant prophylaxis. There seemed to be a tendency to universal prophylaxis with broad spectrum antifungal agents over targeted prophylaxis [89].

The introduction of anti-fungal prophylaxis has reduced rates of invasive fungal infections. In a retrospective single center study of 584 pediatric solid organ recipients, 37% of lung transplanted children received antifungal prophylaxis, mainly voriconazole, a greater percentage than other solid organ recipients. The use of antifungal prophylaxis among all solid organ recipients increased from 7% in 2000–2006 to 11% in 2007–2013, whilst invasive fungal infections decreased from 4% to 1% [90]. A review and meta-analysis of six observational studies comprising 748 adult patients in total suggested that universal antifungal prophylaxis reduced the risk of invasive pulmonary aspergillus infections after lung transplantation [91], although the authors acknowledged a number of limitations such as small and often heterogeneous study populations, single-center and often non-randomized study designs, and different immunosuppressive and prophylactic treatment regimes. It has to be mentioned that another systematic review and meta-analysis of 22 studies (235 patients) neither found a significant reduction of *Aspergillus* spp. colonization nor invasive pulmonary infection, probably due to similar limitations [92].

## 10. Conclusions

With progress in mycological culture protocols and non-culture based, molecular techniques, the range and detection rates of fungi isolated from CF airway samples are expanding. Our knowledge on the clinical relevance of positive cultures is limited, and the epidemiology on fungi in CF is still in its infancy. In the absence of clear consensus criteria for most respiratory fungal conditions and univocal treatment protocols, clinicians are faced with the challenges of distinguishing harmless airway colonization from damaging fungal disease. In the first case, the starting treatment might pose unnecessary burden and potential adverse drug effects to the patient, in the latter, negligence might result in irreversible lung damage. Failure to eradicate certain fungal colonizations, in particular *Lomentospora* and *Scedosporium*, might mean excluding the patient from lung transplantation. We need to better define clinical manifestations of respiratory fungal diseases and identify those patients who will benefit from treatment. We need to investigate the impact of novel CFTR modulating agents on the lung microbiome, and we need to improve and harmonize prophylactic- and treatment strategies for high risk populations (e.g., end stage CF lung disease and CF patients after lung transplantation). After the great achievements of antibiotic treatment of bacterial pulmonary exacerbations, and the introduction of CFTR modulators with disease modifying potential, respiratory fungal infections in CF will be our next challenge in improving the treatment of CF.

## Figures and Tables

**Table 1:** Prevalence of fungi in cystic fibrosis (CF) airway samples.

*Candida* spp.	Ziesing et al., Germany, 2009–2013 [13]	*Candida* spp. 75% *C. albicans* 38% *C. dubliniensis* 12% *C. glabrata* 9% *C. parapsilosis* 3% *C. lusitaniae* 2% *C. krusei* 1%
	Coron et al., France, 2013 [14]	*C. albicans* 58.8%
	Schwarz et al., 9 European Centers, 2011–2016 [15]	*C. albicans* 33.8–77.9% *C. dubliniensis* 0.9–14.6% *C. glabrata* 1.2–7.3% *C. parapsilosis* 1–28% *C. krusei* 0.5–1.1%
	Al Shakirchi et al., Sweden 2000–2015 [16]	*C. albicans* 62% *C. dubliniensis* 11%
*Aspergillus* spp.	Ziesing et al., Germany, 2009–2013 [13]	*Aspergillus* spp. 35% *A. fumigatus* 29%
	Felton et al., UK 2007–2012 [17]	*Aspergillus* spp. 6.5% (2007) *Aspergillus* spp. 13.6 % (2012)
	Engel et al., The Netherlands, 2018/19 [18]	*A. fumigatus* 55.9%
	Coron et al., France, 2013 [14]	*A. fumigatus* 35.4%
	Hong et al., United States, 2017 [19]	*Aspergillus* spp. 40.8%
	Schwarz et al., 9 European Centers, 2011–2016 [15]	*A. fumigatus* 3.9–42.4%
	Al Shakirchi et al., Sweden 2000–2015 [16]	*A. fumigatus* 22%
*Scedosporium/Lomentospora* complex	Ziesing et al., Germany 2009–2013 [13]	4%
	Felton et al., UK 2007–2012, [17]	0.07% (2007) 0.68% (2012)
	Engel et al., The Netherlands, 2018/19 [18]	1.1%
	Hong et al., United States, 2017 [19]	5.2%
	Schwarz et al., 9 European Centers, 2011–2016 [15]	*Scedosporium* spp. 0.1–12.1% *Lomentospora proflificans* 0–3.8%
	Blyth et al., Australia 2008/09 [20]	*Scedosporium* spp. 17.4%
	Sedlacek et al., Germany, 2011 [21]	0–10.5%
	Chen et al., Sweden 2012 [22]	*S. apiospermum*/*S. boydii* 1.2% (culture), 30% (molecular detection)
	De Jong et al., The Netherlands, 2010–2013 [23]	*Scedosporium* spp. 7%
*Exophiala dermatitidis*	Ziesing et al., Germany 2009–2013 [13]	4%
	Felton et al., UK 2012 [17]	0.54%
	Engel et al., The Netherlands, 2018/19 [18]	1.5%
	Hong et al., United States, 2017 [19]	4.7%
	Schwarz et al., 9 European Centers, 2011–2016 [15]	0.2–18.3%
	Chen et al., Sweden 2012 [22]	14.2% (culture), 17% (molecular detection)
	De Jong et al., The Netherlands, 2010–2013 [23]	2%

**Table 2:** Respiratory fungal conditions in cystic fibrosis (CF) lung disease and after lung transplantation.

Colonization with Unclear Clinical Relevance and Without Clinical Symptoms	
Fungal bronchitis	
Fungal pneumonia	
Allergic broncho-pulmonary aspergillosis (APBA)	
Aspergilloma	
Distinct entities in lung transplant recipients:	
	Aspergillus tracheobronchitis (ATB)
	(Bronchiolitis obliterans syndrome (BOS)–*Aspergillus* colonization may be a risk factor)
